# Effects of a complex probiotic preparation, Fengqiang Shengtai and coccidiosis vaccine on the performance and intestinal microbiota of broilers challenged with *Eimeria* spp.

**DOI:** 10.1186/s13071-023-05855-5

**Published:** 2023-07-27

**Authors:** Haiming Cai, Shengjun Luo, Qihong Liu, Qingfeng Zhou, Zhuanqiang Yan, Zhen Kang, Shenquan Liao, Juan Li, Minna Lv, Xuhui Lin, Junjing Hu, Shuilan Yu, Jianfei Zhang, Nanshan Qi, Mingfei Sun

**Affiliations:** 1 Zhaoqing/Maoming Branch Center of Guangdong Laboratory for Lingnan Modern Agricultural Science and Technology, Key Laboratory of Livestock Disease Prevention of Guangdong Province, Key Laboratory of Avian Influenza and Other Major Poultry Diseases Prevention and Control, Ministry of Agriculture and Rural Affairs, Institute of Animal Health, Guangdong Academy of Agricultural Sciences, Jinying Road, Tianhe District, Guangzhou, 510640 Guangdong People’s Republic of China; 2 Jiangsu HFQ Biotechnology Co., Ltd., Haimen, Jiangsu Province People’s Republic of China; 3 Wen’s Group Academy, Wen’s Foodstuffs Group Co., Ltd., Xinxing, Guangdong 527400 People’s Republic of China; 4 Qingdao Vland Biotech Group Co., Ltd., Qingdao, Shandong Province People’s Republic of China; 5 Laboratory of Parasitology, Institute of Animal Health, Guangdong Academy of Agricultural Sciences, Jinying Road, Tianhe District, Guangzhou, 510640 Guangdong People’s Republic of China

**Keywords:** Probiotics, Coccidiosis vaccine, Production performance, Intestinal microbiota, 16S rRNA

## Abstract

**Background:**

Coccidiosis, a prominent intestinal protozoan disease, carries significant economic implications for the poultry industry. The aim of this study was to evaluate the effects of Fengqiang Shengtai (BLES), a probiotics product, and coccidiosis vaccine in modulating the intestinal microbiome and providing insight into mitigating the occurrence and management of avian coccidiosis.

**Methods:**

Broilers included in the study were divided into four pre-treatment groups: the Pre-Con group (commercial diet), Pre-BLES group (BLES supplement), Pre-Vac group (coccidiosis vaccination) and Pre-Vac-BLES group (combined vaccination and BLES). Body weight gain, feed consumption and feed conversion ratio were monitored from age 25 to 55 days. Cecum contents were collected at 8 and 15 days of age for comparative analysis of intestinal microbiomes. In the Pre-BLES and Pre-Vac-BLES groups, probiotics were administered at a dose of 0.01 g per chicken between ages 3 to 6 days and 10–13 days. At 3 days of age, chickens in the Pre-Vac and Pre-Vac-BLES groups were vaccinated with 1700 sporulated oocysts of the live coccidiosis vaccine per chicken. At the age of 25 days, *Eimeria* spp. challenge experiments were performed based on the aforementioned immunization strategy, and the oocysts per gram (OPG) in the feces, intestinal lesion score and intestinal pathological characteristics were evaluated. Specifically, 30 chickens were randomly selected from each group and orally administered 34,000 sporulated oocysts of *Eimeria* spp. per chicken, re-defined as Eimeria group, BLES-Eimeria group, Vac-Eimeria group and Vac-BLES-Eimeria group, respectively. Additionally, 30 chickens were randomly selected from the Pre-Con group and included as negative control without *Eimeria* spp. challenge. Intestinal microbiota was sequenced and analyzed when the broilers were 32 days old.

**Results:**

A significant improvement was observed in body weight gain of the broilers in the Pre-BLES and Pre-Vac-BLES group at 45 days of age. Analysis of the intestinal microbiota revealed a positive correlation between the experimental groups receiving BLES and coccidiosis vaccines at 8 and 15 days of age with the *Enterococcus* genus and *Lachnospiraceae* NK4A136 group, respectively. In addition to the reduced lesion score and OPG values, the combination of coccidiosis vaccine and BLES also reduced the intestinal epithelial abscission induced by coccidiosis vaccines. The results of intestinal microbial function prediction demonstrated that N-glycan biosynthesis and ferroptosis were the prominent signal pathways in the Vac-BLES-Eimeria group.

**Conclusions:**

Taken together, the results of the present study suggest that supplementation of BLES with coccidiosis vaccine represents a promising strategy for improving growth performance, alleviating clinical manifestations and inducing favorable alterations to the intestinal microbiota in broiler chickens affected by coccidiosis.

**Supplementary Information:**

The online version contains supplementary material available at 10.1186/s13071-023-05855-5.

## Background

Intestinal parasitosis poses a significant challenge to the global livestock and poultry industry. The protozoan parasite *Eimeria* spp. in particular is a well-known pathogenic obligate intracellular parasite that can infect livestock species [1]. Avian coccidiosis, caused by the *Eimeria* spp., manifests as an intestinal disease characterized by disruptions in intestinal integrity, impaired digestion and nutrient absorption, compromised production performance and increased susceptibility to bacterial infections [2, 3]. As a consequence of its high occurrence and mortality rates, avian coccidiosis has inflicted substantial economic losses upon the poultry industry, surpassing an annual deficit of US$ 13–14 billion [4]. Chickens are currently known to be susceptible to seven *Eimeria* species, with *E. tenella*, *E. acervulina*, *E. necatrix* and *E. maxima* demonstrating the greatest pathogenicity and highest clinical risk [5]. Therefore, effective prevention and control measures against *Eimeria* infection assume paramount importance in poultry industry nowadays.

Currently, the management of avian coccidiosis primarily relies on the utilization of anti-coccidiosis drugs and live vaccines. However, the employment of anti-coccidial drugs not only impairs the intestinal health and production performance of broilers, but also gives rise to food safety concerns stemming from drug residues [6, 7]. In addition, the long-term efficacy of anti-coccidiosis drugs in preventing and controlling coccidiosis is deemed inadequate due to the emergence of drug-resistant strains of *Eimeira* spp.

Therefore, live vaccines are increasingly used to prevent and control avian coccidiosis, and their safety and reliability have been demonstrated in both laying hens and broilers [8]. However, despite efforts to attenuate their virulence via modifications, live vaccines still retain the potential to hamper poultry production performance and increase the susceptibility of young chickens to secondary infections by other pathogens [9]. Consequently, the focus has shifted towards feed additive products, which have emerged as a popular avenue for avian coccidia prevention and control. Additionally, the development of a coccidiosis vaccine can be expensive, with vaccine efficacy heavily contingent upon the prevalent strains of *Eimeria* present in a specific location and country and on management practices. Therefore, identification of effective strategies to mitigate adverse reactions associated with live vaccines is an urgent problem that requires immediate attention in relation to avian coccidia prevention and control.

Probiotics, as living non-pathogenic bacteria, have the potential to preserve gastrointestinal health, maintain a balanced cecal microbiota and improve poultry production performance. Various probiotics, including *Lactobacillus*, *Pediococcus*, *Enterococcus*, *Bacillus* and *Saccharomyces cerevisiae*, can be employed as additives in poultry diets [10, 11]. The mechanisms of probiotic action are diverse and may encompass the production of antibacterial compounds, the induction of competition for nutrients and the occupation of adhesion sites on host cells. Some studies have explored the supplementation of probiotics such as *Bacillus* and *Clostridium butyricum*, either in water or diets, to mitigate or regulate the adverse effects of coccidia infection in livestock [12–14]. However, it is still unclear whether the combination of live vaccines and probiotics can ameliorate or eliminate the side effects associated with live vaccine in production performance. Furthermore, the effectiveness of this combination in the control of chicken coccidiosis warrants further investigation.

The probiotic product Fengqiang Shengtai feed additive (BLES) is a preparation composed of four live bacterial strains, namely *Bifidobacterium animalis*, *Lactobacillus casei*, *Enterococcus faecalis* and *Saccharomyces cerevisiae*. A previous study demonstrated its positive effects on the growth, health and fecal bacterial flora of neonatal dairy calves [15]. Building upon this prior research, we hypothesized that the combined administration of BLES and live vaccine in feed could improve the production performance of broilers and effectively prevent and control avian coccidiosis. Therefore, this study aimed to evaluate the individual and combined effects of probiotics and live vaccine on broiler production performance and the prevention of chicken coccidia infection. Furthermore, we also explored the potential intestinal barrier repair effects of the aforementioned treatment by analyzing the changes in intestinal microbiota composition and function.

## Methods

### Animals, parasites and probiotics

A total of 1000 1-day-old Mahuang broilers were purchased from Chaozhou breeding farm, and meticulous sterilization measures were employed using a Blaze disinfection gun to ensure a coccidia-free environment. Animal trials were conducted in strict accordance with the guidelines of the animal care and use Committee of the Institute of Animal Health, Guangdong Academy of Agricultural Sciences (No. PT-2020030).

The avian coccidia strains utilized in this study were maintained in the parasitology laboratory of the Institute of Animal Health, Guangdong Academy of Agricultural Sciences. The sporulation and purification processes of chicken coccidia oocysts were performed according to the methodologies previously outlined in [16]. The tetravalent live vaccine against avian coccidiosis, consisting of the *E. tenella* ETGZ strain, *E. necatrix* ENHZ strain, *E. acervulina* EAGZ strain and *E. maxima* EMPY strain, was prepared by the parasitology laboratory of the Institute of Animal Health, Guangdong Academy of Agricultural Sciences. The vaccine was stored in a 2.5% potassium dichromate solution at 4 °C in the form of sporulated oocysts for subsequent use.

The probiotic product BLES employed in this trial consisted of the four live bacterial strains listed above at the following concentrations: *B. animalis* (1.0 × 10^7^ colony-forming unit [CFU]/g); *L. casei* (1.0 × 10^7^ CFU/g); *E. faecalis* (1.0 × 10^6^ CFU/g); *Saccharomyces cerevisiae* (1.0 × 10^6^ CFU/g). The BLES product was procured from Jiangsu HFQ Biotechnology Co., Ltd. (Haimen, China)

### Pre-treatment period

A total of 990 1-day-old broiler birds were randomly divided into groups prior to treatment: the control group (Pre-Con group [commercial diet], *n* = 270); the Vac group (Pre-Vac group [coccidiosis vaccination], *n* = 240); the BLES group (Pre-BLES group [BLES supplement], *n* = 240); and the Vac-BLES group (Pre-Vac-BLES group [combined vaccination and BLES], *n* = 240). Three floor pens were utilized in each group, with 90 broiler birds randomly allocated to each pen in the control group, and 80 broiler birds assigned to each pen in the three treatment groups. The final bird density in each pen was maintained at 12 birds/m^2^. Feed and water were provided ad libitum from 1 to 55 days of age. In both the Pre-Vac and the Pre-Vac-BLES groups, chickens were vaccinated with live coccidiosis vaccine (2004003; QiluTsingta Biopharmaceutical Co., Ltd.) containing 1700 sporulated oocysts per chicken using sodium carboxymethyl cellulose (CMC; Dai-ichi Kogyo Seiyaku Co., Ltd., Tokyo, Japan; 0.0625% m/V) dissolved in water at 3 days of age. In both the Pre-BLES group and the Pre-Vac-BLES groups, chickens were fed with probiotics at a dose of 0.01 g per chicken during two periods: 3–6 days of age and 10–13 days of age.

### *Eimeria* spp. challenge

At the age of 25 days, 30 birds (10 birds per pen) were randomly selected from each pre-treatment group for *Eimeria* spp. challenge, and an additional 30 birds (10 birds per pen) from the Pre-Con group were selected for the no-challenge control. These birds were subjected to *Eimeria spp.* challenge and were subsequently referred to as the Eimeria group, BLES-Eimeria group, Vac-Eimeria group, Vac-BLES-Eimeria group and the control group, respectively. The remaining birds in each group were subjected to growth performance monitoring during the finisher phase and retained the same group names as in the pre-treatment experiment. All birds were individually housed in separate steel cages and had free access to fresh, clean drinking water and feed throughout the experimental period. Except for the control group, birds in all the challenge groups were orally challenged with *Eimeria* spp. at a dose of 34,000 sporulated oocysts per chicken. The specific composition of the challenge dose included *E. tenella* (10,000 sporulated oocysts), *E. necatrix* (4000 sporulated oocysts), *E. acervuline* (10,000 sporulated oocysts) and *E. maxima* (10,000 sporulated oocysts). The birds in the control group were administered the same volume of phosphate-buffered saline.

### Performance indicators

During the pre-treatment phase, chickens in the Pre-Con group, Pre-Vac group, Pre-BLES group and Pre-Vac-BELS group were monitored closely for production performance parameters at 25, 35, 45 and 55 days of age. The production performance parameters assessed included body weight gain (BWG), feed intake (FI) and feed conversion ratio (FCR). Each broiler was individually weighed at the beginning and the end of each experimental period. The average BWG (g/bird) in each experimental stage was calculated by subtracting the initial body weight from the final body weight. To calculate the overall FI of each broiler (g/bird) throughout each experimental phase, the amount of feed offered was divided by the number of broilers that had access to the same feeder. The FCR (g/g BWG) was calculated by dividing the FI by the BWG obtained during each experimental period. All data acquisition and calculations were conducted following the methodology established in a previous study [17].

### Clinical pathological characteristics

In the challenge experiment, fecal samples were collected from the Eimeria group, BLES-Eimeria group, Vac-Eimeria group, Vac-BLES-Eimeria group and control group at the age of 30–32 days to calculate the number of oocysts per gram (OPG) using the McMaster method [18]. At the age of 32 days, chickens were euthanized, and two duplicate samples of the duodenum, jejunum and cecum were collected from each chicken. One of the two duplicate intestinal tissues were fixed in 10% formalin, prepared on slides for the histological studies and subsequently stained with hematoxylin and eosin (H&E). Changes in the morphology of the intestines were analyzed based on the histopathological sections. The duplicate set of intestinal samples was scored for lesions, with a score of 0 indicating the absence of any obvious lesions; a score of 1 indicating the presence of 1–4 petechiae/cm^2^ on serosal membranes; a score of 2 indicating the presence of 5–10 petechiae/cm^2^ on the serosal membrane; and a score of 3 indicating the presence of ≥ 11 petechiae/cm^2^ on the serosal membrane[19]. OPG counting, H&E staining and lesion scoring were performed on 10 randomly selected samples from each experimental group.

### DNA extraction, amplicon generation and Illumina MiSeq sequencing

In the Pre-treatment experiment, four chickens were randomly selected from each of the three replicate pens in each group at the age of 8 and 15 days for collection and analysis of cecum contents. Similarly, in the challenge experiment, an equal number of chickens at age of 32 days from each group were randomly selected for collection and analysis of cecum contents. The cecum contents were then stored in a − 80 °C refrigerator for subsequent DNA isolation.

For DNA isolation, microbial genomic DNA of the cecum contents was extracted using the E.Z.N.A.® Stool DNA Kit (Omega Bio-Tek, Norcross, GA, USA). In accordance with the manufacturer’s recommendations, the concentration and integrity of the extracted DNA were tested to ensure the high quality of the sample. The 16S rDNA V3-V4 hypervariable region was then amplified using the primer pair 338F (5′-ACTCCTACGGGAGGCAGCAG-3′) and 806R (5′-GGACTACHVGGGTWTCTAAT-3′). Amplification of the 16S ribosomal RNA (rRNA) gene was carried out as follows: initial denaturation at 95 °C for 3 min; 27 cycles of denaturation at 95 °C for 30 s, annealing at 55 °C for 30 s; followed by 45 s of extension at 72 °C, culminating at 4 °C. The PCR mixture contained 5× *TransStart* FastPfu buffer (4 μl), 2.5 mM dNTPs (2 μl), forward primer (5 μM; 0.8 μl), reverse primer (5 μM; 0.8 μl), *TransStart* FastPfu DNA Polymerase (0.4 μl) and template DNA (10 ng), with ddH_2_O added to achieve a reaction volume of 20 μl. All PCR reactions were conducted in triplicate. The AxyPrep DNA Gel Extraction Kit (Axygen Biosciences, Union City, CA, USA) was used to purify the PCR product, which was then quantified using a Quantus™ Fluorometer (Promega, Madison, WI, USA). Purified amplicons were pooled in equimolar concentrations and paired-end sequenced on an Illumina MiSeq PE300 platform/NovaSeq PE250 platform (Illumina, San Diego, CA, USA) according to the standard protocols by Majorbio Bio-Pharm Technology Co. Ltd. (Shanghai, China).

### Sequence processing

All of the raw data were processed by joint cleaning, low-quality data cleaning and data noise reduction via Quantitative Insights Into Microbial Ecology 2 (QIIME2) software. Among these, sequence assembly and low-quality data cleaning were performed by the QIIME2 cutadapt plugin, while the data noise was eliminated by the QIIME2 DATA2 plugin, with the following parameters: “-p-trunc-len-f 0, -p-trunc-len-r 0, -p-n-threads 40”. Afterwards, each 16S rRNA gene was classified and annotated with SILVA 132 ribosomal RNA databases at a 99% shared identity using the “qiime feature-classifier classify-sklearn” command. The retained amplicon sequence variants (ASVs) were then aligned to construct a phylogenetic tree with the “qiime phylogeny align-to-tree-mafft-fasttree” command, following which alpha diversity, beta (β) diversity, microbiota composition and microbiota function prediction were analyzed using RStudio, version 1.4.1717 software. The representative sequence, feature abundance table, taxonomic information table, phylogenetic tree and metadata were imported as “meco_physeq” R objects using qiime2meco in the R package microeco (v.0.7.1). The “meco_physeq” was then converted to “physeq” R objects using meco2phyloseq function. Alpha diversity of intestinal microbiota was characterized using the Chao1 estimator, observed OTUs and Shannon diversity indices, using get_rarecurve and get_alphaindex in R package MicrobiotaProcess (v.3.6.0). Based on the evenly rarefied OUT abundance table, Bray–Curtis distance and Euclidean distance were used to estimate the β-diversity, which measured the difference in community structure between samples, using get_pcoa in the MicrobiotaProcess package. Subsequently, a principal coordinate analysis (PCoA) plot was built using ggordpoint in the MicrobiotaProcess package. The abundances at different taxonomic levels were compared by visualizion with bar plots using trans_abund and in the microeco package. The shared or particular taxa among groups were analyzed using trans_venn in the microeco package. In the pre-treatment experiments, differential abundance at genus level among each group was analyzed by trans_diff class with the random forest method and non-parametric Kruskal–Wallis tests to determine significant differences in microbial taxa using the microeco package. The biomarker taxa in the pre-treament groups at 15 days of age were identified by the linear discriminant analysis (LDA) effect size (LEfSe) algorithm in the trans_diff function with parameters: method = “lefse”, LDA_score = 2, alpha = 0.05. In the *Eimeria* spp. challenge experiment, heatmap analysis of the top 50 genera was performed using plot_taxa_heatmap in the R package microbiomeutilities (v.1.00.16). The significant difference of microbial taxa between groups was analyzed by DESeq2 with phyloseq_to_deseq2 and DESeq under parameters: test = "Wald", fitType = "parametric" in R package phyloseq (v.1.36.0) and the plot was built using ggplot2 (v.3.3.5). The microbial functional prediction was conducted using the cal_tax4fun2 function. The metabolic pathway abundance was calculated by Tax4Fun2 using normalized 16S rRNA gene copy number, and subsequently categorized by function using the Kyoto Encyclopedia of Genes and Genomes (KEGG) Orthology (KO) database; the results were visualized using ggplot2. Otherwise, differences in molecular functions were analyzed by the LEfSe with default settings (alpha value for the Kruskal–Wallis test among classes = 0.05; logarithmic LDA score for discriminative features = 2) in diff_analysis function and the plot was built with ggdiffbox and ggdifftaxbar function in the MicrobiotaProcess package.

### Statistical analysis

In this study, analysis of variance (ANOVA) followed by Tukey's honest significant differences (HSD) post hoc test were employed for the analysis of differences in production performance or clinical lesion index following the challenge. Data analysis and visualization were completed using the SPSS software package version 19 (SPSS IBM Corp., Armonk, NY, USA) and GraphPad Prism software package version 6 (GraphPad Software, San Diego, CA, USA). The analyzed data were presented as the mean ± standard error of the mean (SEM). Statistical significance in comparison to the control group was set at *P* < 0.05 and *P* < 0.01.

## Results

### Effect of BLES or BLES combined with avian coccidiosis vaccine on broiler production performance

**Fig. 1:**
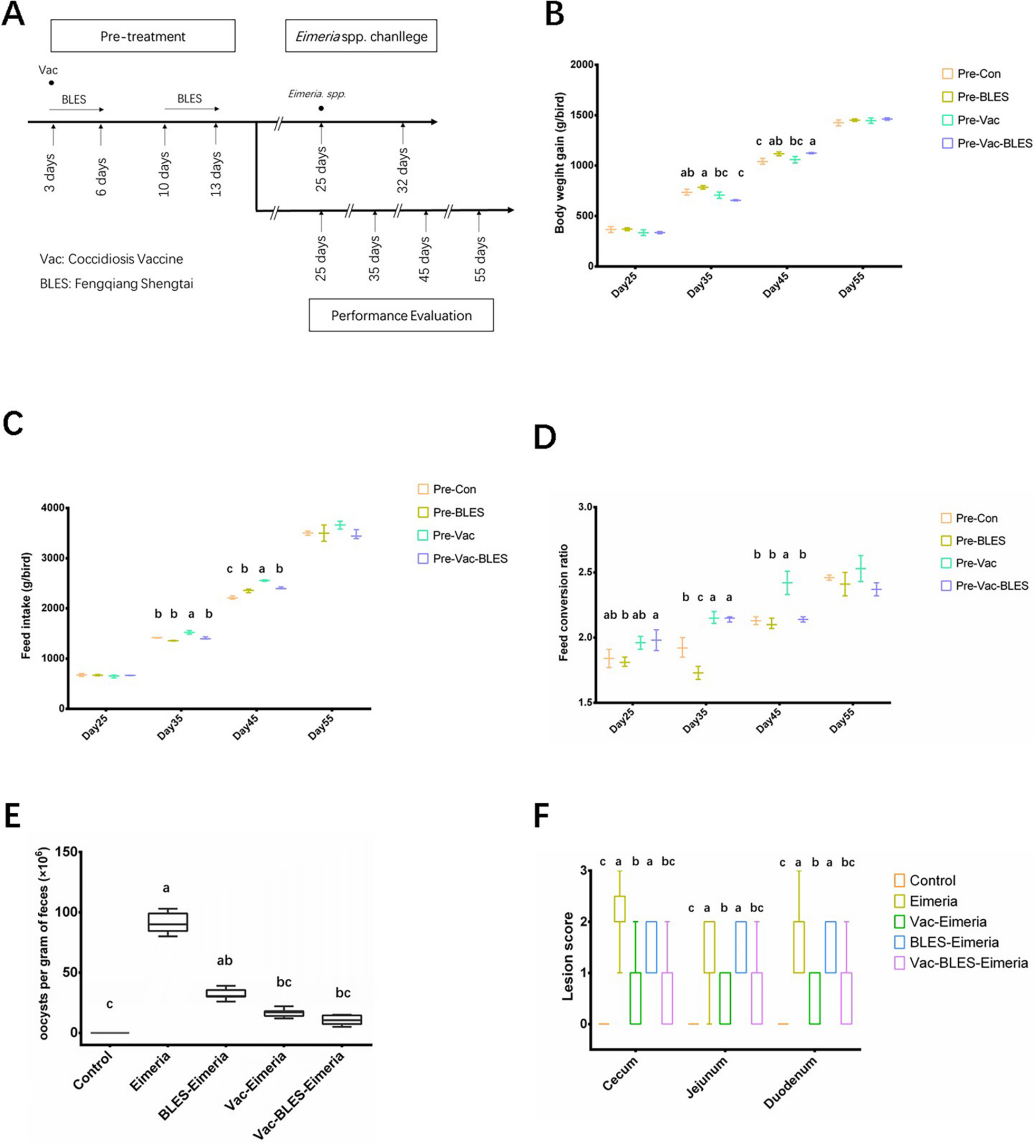
Technical aspects of the study and the related animal clinical detection indicators. **A** The study was primarily divided into a pre-treatment and *Eimeria* challenge experiment, respectively. In the pre-treatment experiment, a control group and three treatment groups were set up, with the latter including immunization with avian coccidiosis vaccine (Pre-Vac group), supplementation to feed of the probiotic product Fengqiang Shengtai quadruple probiotic preparation (Pre-BLES group) and combined administration of Vac and BLES (Pre-Vac-BLES group). In the chicken coccidia vaccine immune experiment, production performance was measured in broilers at different time points between the age of 25 and 55 days, and the intestinal contents were collected at the age of 8 and 15 days for intestinal microbial community sequencing. In the challenge experiment, based on the immune test, all groups were challenged with *Eimeria* spp. at 25 days of age. After *Eimeria* spp. challenge, the Pre-Vac, Pre-BLES and Pre-Vac-BLES groups were redefined and referred to as the Vac-Eimeria, BLES-*Eimeria* and Vac-BLES-Eimeria groups, respectively. At the same time, the *Eimeria* group that was infected with *Eimeria* spp. and the control group that was not infected were also set up. In the challenge experiment, fecal oocyst counts, lesion scoring and intestinal pathological section detection were performed. Accordingly, the intestinal contents were collected for bacterial population sequencing when broilers were 32 days old. **B**–**D** Production performance of the broilers after immune treatment. **E**, **F** Clinical pathological parameters of the broilers after *Eimeria* challenge: **E** results of the oocytes per gram statistics; **F** scoring results on pathological changes in the different intestinal segments. Analysis of the difference in results was performed according to different ages. Means denoted by a different superscript letter (a, b) within a variable are significantly different at *P* < 0.05. Con, Control group; Pre, Pre-treatment

The technical roadmap was depicted in Fig. 1A, and the results of production performance were presented in Fig. 1B–D.

Following the immune treatment, the production performance of chickens was monitored at 25, 35, 45 and 55 days of age.

A two-way multivariate analysis of variance was employed to investigate the interaction effects of BLES pre-treatment and tetravalent live vaccine immune on production performance, and the results are presented in Additional file 1: Table S1. The interaction effect of the BLES pre-treatment and tetravalent live vaccine immune treatment had a significant influence on BWG (*F* = 266.260, partial *η*^2^ = 0.987, *P* < 0.05). Similarly, for FCR and FI, the individual interaction effects of BLES pre-treatment and tetravalent live vaccine immune treatment were statistically significant, with *P*-values of 0.007 and 0.001, respectively. ANOVA was conducted to analyze inter-group differences in different immunization strategies, revealing that there was no significant difference in both body weight or FI of broilers at the age of 25 days (*P* > 0.05, compared with Pre-Con group) (Fig. 1B, C). However, the FCR in the Pre-BLES group was significantly reduced in the growth period at the age of 25 days in comparison with that of the Pre-Con group (*P* < 0.05) (Fig. 1D). Furthermore, at the age of 35 and 45 days in the finisher period, immunization with avian coccidiosis vaccine alone (Pre-Vac group) resulted in a significant increase in the FI value and FCR value compared with those of the Pre-Con group (*P* < 0.05). However, feed consumption from 35 to 45-days was significantly reduced in the Pre-Vac-BLES group and Pre-BLES group in comparison with that in the Pre-Vac group, leading to a decreased FCR value (*P* < 0.05). These findings revealed that the combined treatment of BLES and vaccination exerted a significant effect on FCR (*P* < 0.05), suggesting that BLES can augment the impact of vaccination in reducing the FCR value. Therefore, the administration of BLES alone or in combination with avian coccidiosis vaccine can confer advantages to the growth performance of broiler chickens.

### Effect of BLES and vaccine pre-treatment on clinical symptoms and pathological characteristics of broilers after *Eimeria* spp. infection

To investigate the effect of BLES and vaccine pre-treatment on the clinical symptoms and pathological characteristics of broilers after *Eimeria* spp. infection, chickens chosen randomly from each pre-treatment group were re-defined as the control group (no *Eimeria* spp. challenge) and four *Eimeria* spp. challenge groups: the Eimeria group, Vac-Eimeria group, BLES-Eimeria group and Vac-BLES-Eimeria group. The results of the OPG test conducted in the challenge experiment demonstrated that OPG number increased significantly in all the *Eimeria* spp. challenge groups compared with the control group (*P* < 0.05) (Fig. 1E). Conversely, the Vac-Eimeria group and the Vac-BLES-Eimeria group exhibited a significant reduction in the OPG value when compared to Eimeria group (*P* < 0.05).

**Fig. 2:**
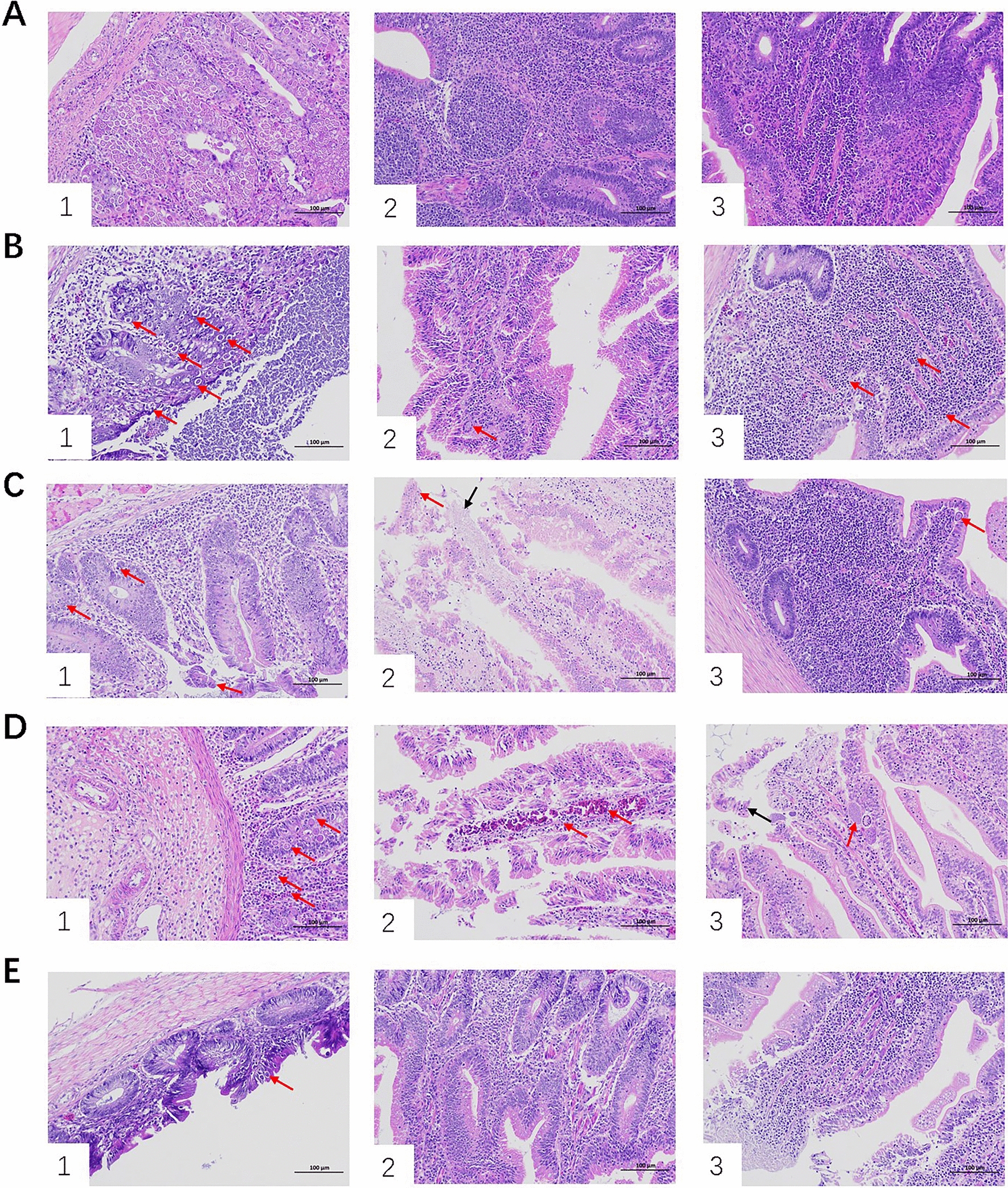
Intestinal histopathological sections from different treatment groups in the challenge experiment. **A**–**E** Results of the intestinal histopathological changes in the control group (**A**), VAC-Eimeria group (**B**), Eimeria group (**C**), BLES-Eimeria group (**D**) and VAC-BLES-Eimeria group (**E**) in the jejunum (*1*), cecum (*2*) and duodenum (*3*) tissues. Scale bar: 100 μm. BLES, Fengqiang Shengtai quadruple probiotic preparation (*Bifidobacterium animalis*, *Lactobacillus casei*, *Enterococcus faecalis* and *Saccharomyces cerevisiae*); Vac, vaccine against avian coccidiosis

The results of intestinal lesion scoring showed no significant difference in the lesion scores among the cecum, jejunum and duodenum between the Eimeria group and the BLES-Eimeria group (Fig. 1F). However, the intestinal lesion scores were significantly reduced in both the Vac-Eimeria group and Vac-BLES-Eimeria group compared to the Eimeria group (*P* < 0.05). We also studied the pathological sections of the three intestinal segments of each challenge test group (Fig. 2). No histopathological changes were observed in the three intestinal segments of the control group (Fig. 2A). In the Eimeria group and the BLES-Eimeria group, coccidian oocysts were observed to be distributed throughout all three intestinal segments, with the jejunum exhibiting the highest abundance of oocysts (Fig. 2B). In the Vac-Eimeria group, the distribution of coccidian oocysts was scattered, and epithelial shedding was observed in the cecum. In the Vac-BLES-Eimeria group, oocysts were only observed in the jejunum, while the epithelium remained intact. These results indicate that the avian coccidiosis vaccine had a significant effect in reducing the clinical and pathological features associated with chicken coccidia infection, and that the addition of BLES prevented epithelial cell shedding.

### Alpha and beta diversity of intestinal microbial communities in BLES and vaccine pre-treatment study or *Eimeria* spp. challenge study

The 16s rDNA V3-V4 region of the intestinal microbiota from 52 experimental animals was sequenced, resulting in a total of 2,908,968 reads (median number of reads per sample: 55,182 [range 36,401–74,261[). Raw data was subjected to the QIIME2 DATA2 data analysis process, including clustering, and taxonomic classification was assigned using the SILVA SSURefNR99 database (release 132). After normalization, a total of 935 ASVs were identified. The alpha diversity of the microbial community was assessed using the Chao1 index, the number of ASVs observed and Shannon's sparse curve (Additional file 1: Figure S1A). The results revealed that 8416 sequences per sample are sufficient to capture the alpha diversity of the microbial community investigated in this study.

Additional alpha diversity analysis was performed on the intestinal microbial community of broilers from both the immune pre-treatment groups and the challenge treatment groups. The Chao1 index, Shannon index and Simpson index were analyzed to detect differences among groups at 8 days (Additional file 1: Figure S1B), 15 days (Additional file 1: Figure S1C) and 32 days of age (Additional file 1: Figure S1D), respectively. No significant difference was observed; however, the mean (± SEM) Chao1 index in the challenge experiment (166.92 ± 64.97) was higher than that in broilers at both the 8-day-old stage (118.44 ± 30) and 15-day-old stage (152.52 ± 73.90) of the immune experiment. Additionally, the mean (± SEM) values of the Shannon index and the inverse Simpson's index in the challenge experiment were 3.54 ± 0.84 and 0.91 ± 0.07, respectively, which were slightly higher than those at the 15-day-old stage (Shannon index: 3.29 ± 1.03; inverse Simpson's index: 0.86 ± 0.14) of the immune experiment. Taken together, these results indicate that the richness and diversity of the intestinal microbial community had increased at the end of the challenge experiment, suggesting that the coccidia infection may affect the intestinal community structure of the chicken formed through the immune treatment.

The beta diversity of the microbial communities was also analyzed using Bray–Curtis and Euclidean distances (Additional file 1: Figure S2). We noted that although each test group overlapped on the coordinate axis, the clusters were clearly defined and the microbial communities in each group were clearly separated. PCoA of the ASVs revealed that ASV_233 (*Eubacterium coprostanoligenes* group), ASV_673 (*Enterococcus*) and ASV_315 (*Subdoligranulum*) were the most influential ASVs in the entire community in broiler from both the immunization experiment and the challenge experiment. Therefore, both the immune stage and the challenge stage exhibited distinct characteristics in terms of microbial community diversity. These distinctive characteristics might provide insights into the underlying mechanism of the immune strategy.

### Effect of BLES and avian coccidiosis vaccine on the intestinal microbial structure of broilers

**Fig. 3:**
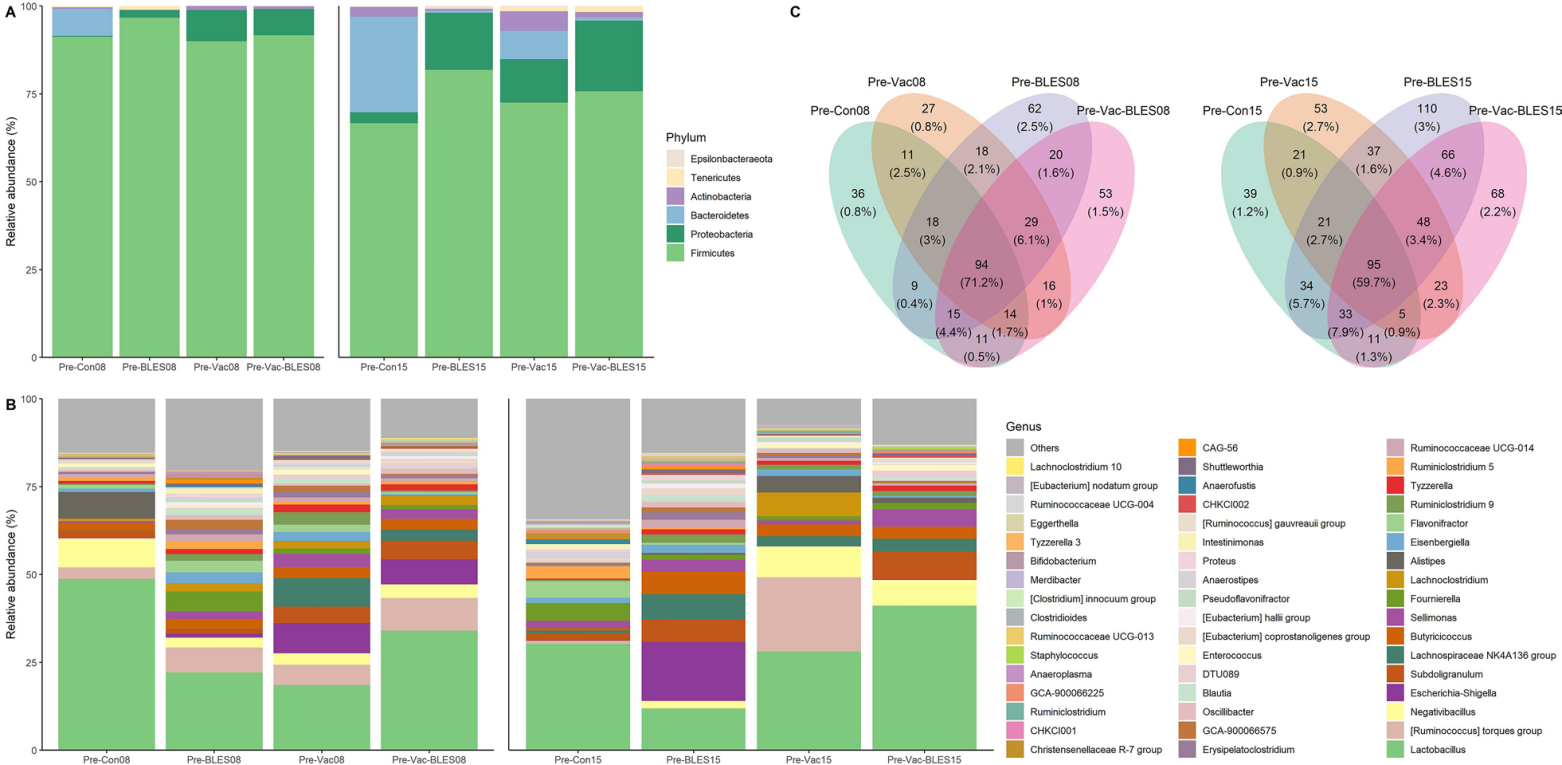
Composition of the intestinal microbiota in each subgroup of immunization experiment. The microbial community composition of the intestinal contents in the treatment groups at 8 and 15 days of age are described at the phylum level (**A**) and the genus level (**B**) using the trans_abund and in microeco package (v.0.7.1). **C** ASV taxa in each test group at 8 and 15 days of age are shown as unique and shared components using trans_venn function. 08, 15, Life stage (in number of days) at which analysis was performed; BLES, Fengqiang Shengtai quadruple probiotic preparation; Con, control group; Pre, pre-treatment groups; Vac, vaccine against avian coccidiosis

An analysis of the relative abundance of microbiota in intestinal scrapings was completed at the 8-day-old stage and 15-day-old stage of the immunization experiment at both the phylum and genus levels. The most abundant phyla of each group at these two age stages were *Actinobacteria*, *Bacteroidetes*, *Firmicutes* and *Proteobacteria*, among which *Firmicutes* was the dominant phylum (8-day age: 92.38%; 15-day age: 74.20%) (Fig. 3A). Compared with the Pre-Con group, at the 8-day-old stage the other treatment groups had a higher relative abundance of *Proteobacteria*; however, at the 15-day-old stage, the relative abundance of *Proteobacteria* in the other experimental groups had decreased sharply, and the relative abundance of *Bacteroidetes* had increased sharply, with the exception of the control group. At the same time, we analyzed the bacterial community structure at the bacterial genus level in the immune experiment (Fig. 3B). All treatment groups at the age of 8 and 15 days displayed high abundances of the *Lactobacillus* and the *Ruminococcus torques* group. We also constructed a Venn diagram for the overlap of ASVs in the immunization experiment between the 8-day-old stage and the 15-day-old stage (Fig. 3C). The results showed that there were 94 (71.2%) and 95 (59.7%) ASVs unique to the 8-day-old stage and the 15-day-old stage, respectively, with the Pre-BLES-Vac group having 53 (1.5%) and 68 (2.2%) unique ASVs, respectively.

**Fig. 4:**
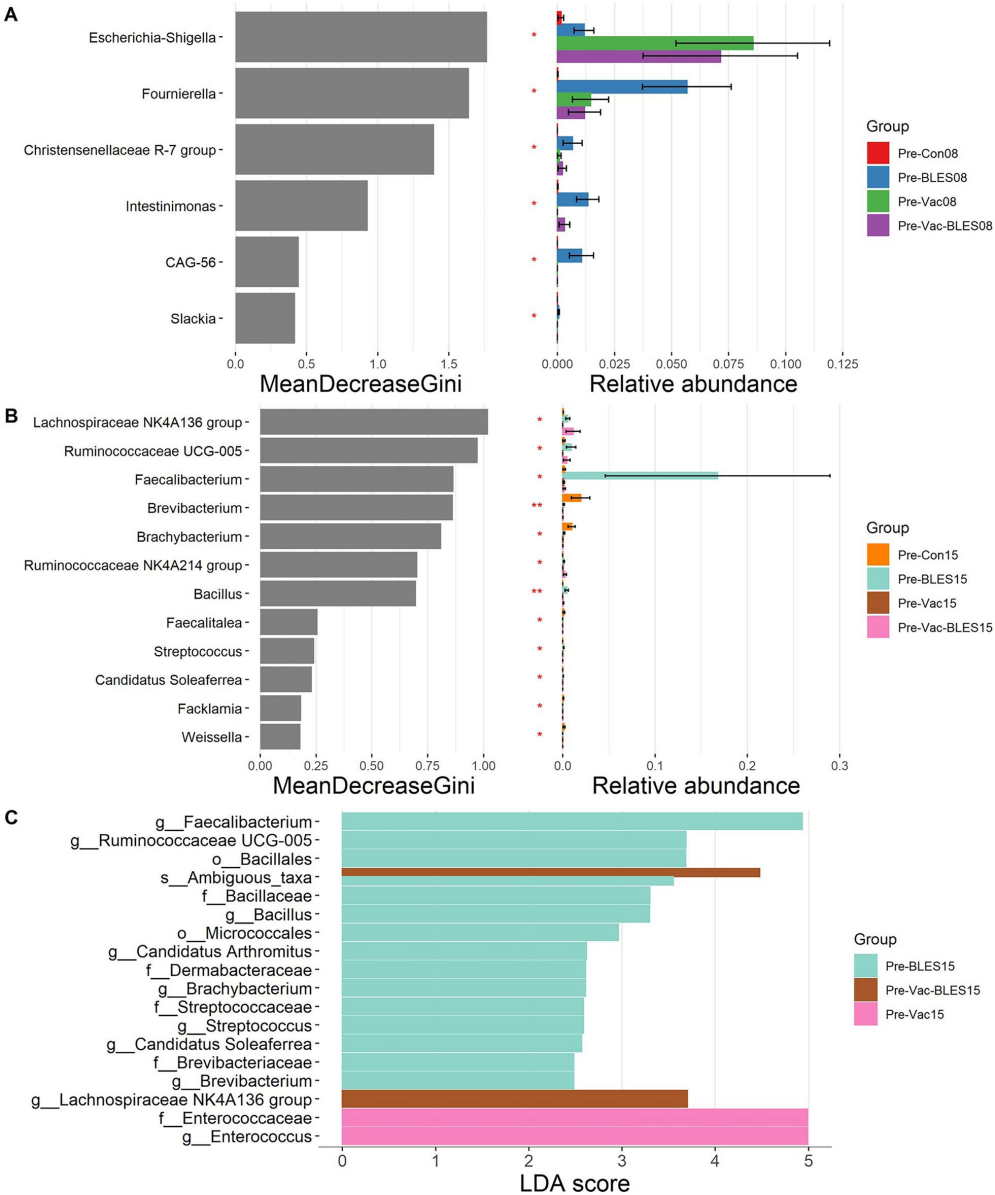
Differential analysis of the intestinal bacterial cohorts between the two phases of the pre-immune experiment. Random forests were sampled to evaluate significant discriminant features from both phases at the age of 8 days (**A**) and 15 days (**B**) using the trans_diff function in R package microeco. (**C**) A LDA was performed for 15 days old using LEfSe. The length of the bar represents the log10 transformed LDA score, indicated by the vertical dashed line. The threshold of the log LDA score for discriminating features is set at 2.0. 08, 15, Life stage (in number of days) at which analysis was performed; BLES, Fengqiang Shengtai quadruple probiotic preparation; CAG-56, * Firmicutes* bacterium CAG-56; Con, control group; Gini, Gini index; LDA, linear discriminant analysis; LEfSe, linear discriminant analysis effect size analysis; Pre, pre-treatment groups; Vac, vaccine against avian coccidiosis

In order to characterize the microbial compositions of each test group in the immunization phase, we applied the random forest classifier to further determine the key distinguishing genera. At the 8-day-old stage, six genera, including *Escherichia-Shigella*, *Foumierela*, *Christensenellacease* R-7 group, *Intestinimonas*,* Firmicutes* bacterium CAG-56 and *Slackia*, showed significant dynamic changes (*P* < 0.05) (Fig. 4A). At the 15-day-old stage, significant dynamic changes (*P* < 0.05) were observed in the abundance of 12 bacterial genera, including *Lachnospiraceae* NK4A136 group, *Ruminococcaceae* UCG-005, *Faecalibacterium*, *Brevibacterium*, *Brachybacterium*, *Ruminococcceae* NK4A214 group, *Bacillus, Faecalitalea*, *Strptococcus*, *Candidatus Soleaferrea*, *Facklamia* and *Weissella* (Fig. 4B). In addition, the Pre-BLES, Pre-Vac-BLES and Pre-Vac groups at the 15-day-old stage were subjected to LEfSe analysis (LDA > 2, *P* value < 0.05). The results showed that in the Pre-BLES group, several distinctive ASVs were mainly concentrated in *Faecalibacterium*, *Candiatus Soleaferrea*, *Candidatus Arthromitus*,* Ruminococcaceae* UCG-005, *Streptococcus* and *Bacillus* within the *Firmicutes* phylum, and in *Brachybacterium* within the *Actinobacteria* phylum (Fig. 4C). However, the Pre-Vac group and Pre-BLES-Vac group showed positive correlations primarily with *Enterococcus* genus and the *Lachnospiraceae* NK4A136 group within the *Firmicutes* phylum.

### Alterations in intestinal microbial composition in BLES and vaccine pre-treatment broilers following the *Eimeria* spp. challenge

**Fig. 5:**
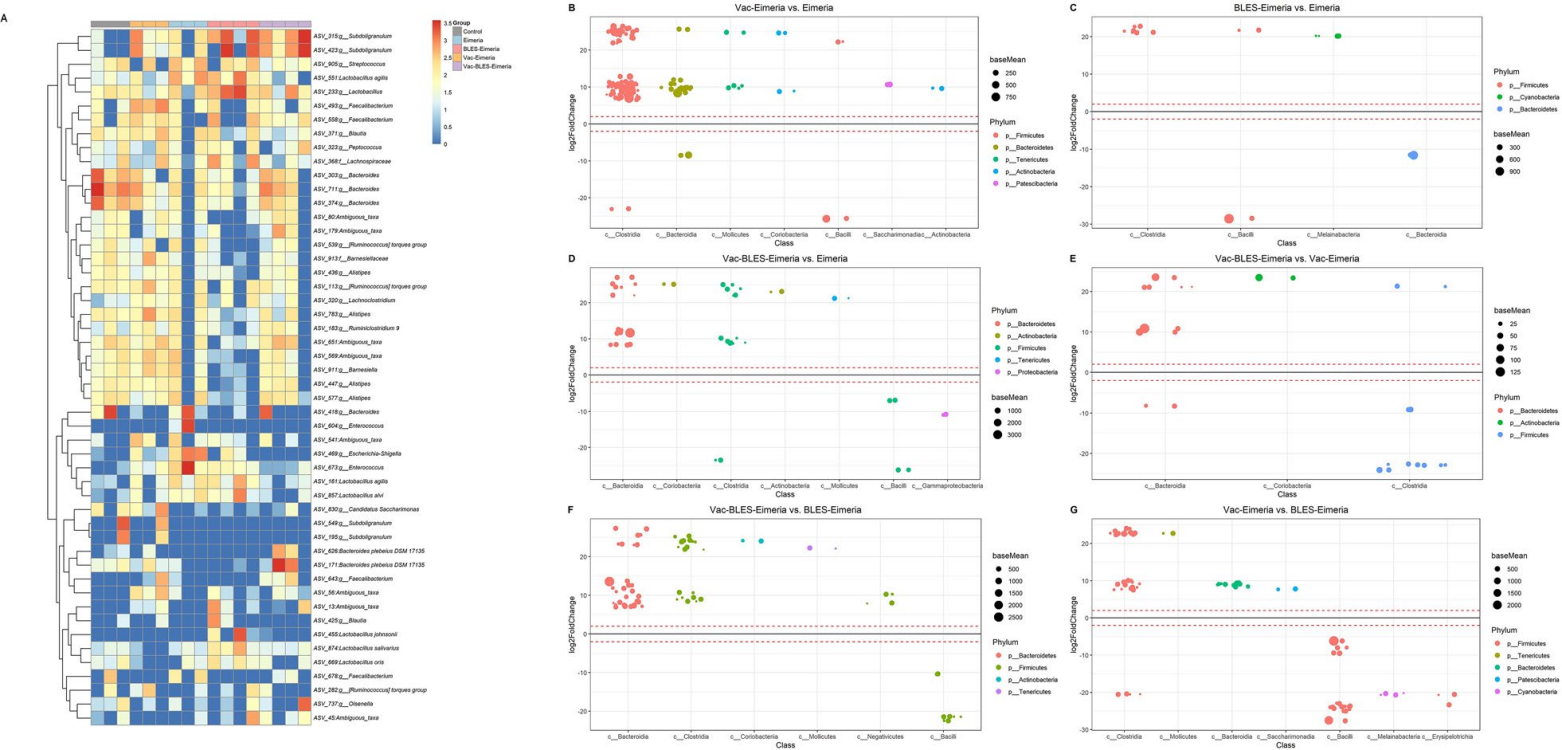
Clustering and difference analysis results at the ASV level of each group in challenge experiment. **a** The top 50 ASVs in terms of relative abundance were selected for cluster analysis, and a pairwise difference analysis was sequentially conducted with plot_taxa_heatmap function in R package microbiomeutilities (v.1.00.16). **b**–**g** Different microbial taxa between experimental groups are shown using phyloseq_to_deseq2 and DESeq function in R package phyloseq (v.1.36.0). The log2FoldChange setting is > 2 and the alpha setting is 0.05. In the case of significantly different levels of ASV abundances, classification was completed at the phylum and class levels. ASVs, Amplicon sequence variants

All treatment groups in the challenge experiment exhibited a diverse species composition (Fig. 5). The top 50 most abundant ASVs in the form of heatmap were mainly distributed in multiple genera, among which *Lactobacillus* (ASV_161, ASV_233, ASV_455, ASV_551, ASV_669, ASV_857, ASV_874) and *Bacteroides* (ASV_171, ASV_303, ASV_374, ASV_418, ASV_626, ASV_711) had the most abundant (Fig. 5A). The heatmap also show significant differences in the abundance of the top 50 ASVs among the different treatment groups.

In order to further analyze the significant changes in the intestinal microbial community structure of the chicken in the challenge experiment, we completed a DESeq analysis between the different treatment groups (parameters: log2FoldChange > 2, alpha = 0.05). Compared to the Eimeria group: (1) in the Vac-Eimeria group, the abundance of 68 ASVs increased and that of four ASVs significantly decreased (*P* < 0.05) (Fig. 5B); (ii) in the BLES-Eimeria group, only seven ASVs were more abundant, and the abundance of two ASVs significantly decreased (*P* < 0.05) (Fig. 5C); (iii) in the Vac-BLES-Eimeria group, 19 ASVs were more abundant, and four ASVs were less abundant (Fig. 5D); moreover, the Vac-BLES-Eimeria group had an overall higher abundance of ASVs compared to the Eimeria group. After performing an overlap analysis of the high-abundance ASVs in the Vac-BLES-Eimeria group with those in the BLES-Eimeria group and Vac-Eimeria group, 13 unique ASVs were identified in the Vac-BLES-Eimeria group. These 13 ASVs were primarily concentrated in the genera *Olsenella*, *Barnesiella*, *Bacteroides*, *Subdoligranulum*, *Anaerotruncus* and *Blautia*. In addition, the low-abundance ASVs in the Vac-BLES-Eimeria group differed from those in the BLES-Eimeria group and the Vac-Eimeria group when compared with the Eimeria group. Only two ASVs, from *Escherichia Shigella* and the *Enterococcus*, were unique to the Vac-BLES-Eimeria group.

We also analyzed the differences in ASV abundance between the BLES-Eimeria group and the Vac-Eimeria group and Vac-BLES-Eimeria group, respectively. Compared to the Vac-Eimeria group: (i) in the Vac-BLES-Eimeria group, seven ASVs exhibited a significantly higher abundance and six ASVs showed a significantly lower abundance (*P* < 0.05) (Fig. 5E); in the Vac-BLES-Eimeria group, 31 ASVs showed a significantly higher abundance and four ASVs exhibited a lower abundance (Fig. 5F). This study further analyzed the differences in ASV abundance between the BLES-Eimeria group and the Vac-Eimeria group. The results revealed that in the BLES-Eimeria group 16 ASVs retained a higher abundance and 24 ASVs had a lower abundance (Fig. 5G).

### Predicted functions of intestinal microbiota

**Fig. 6:**
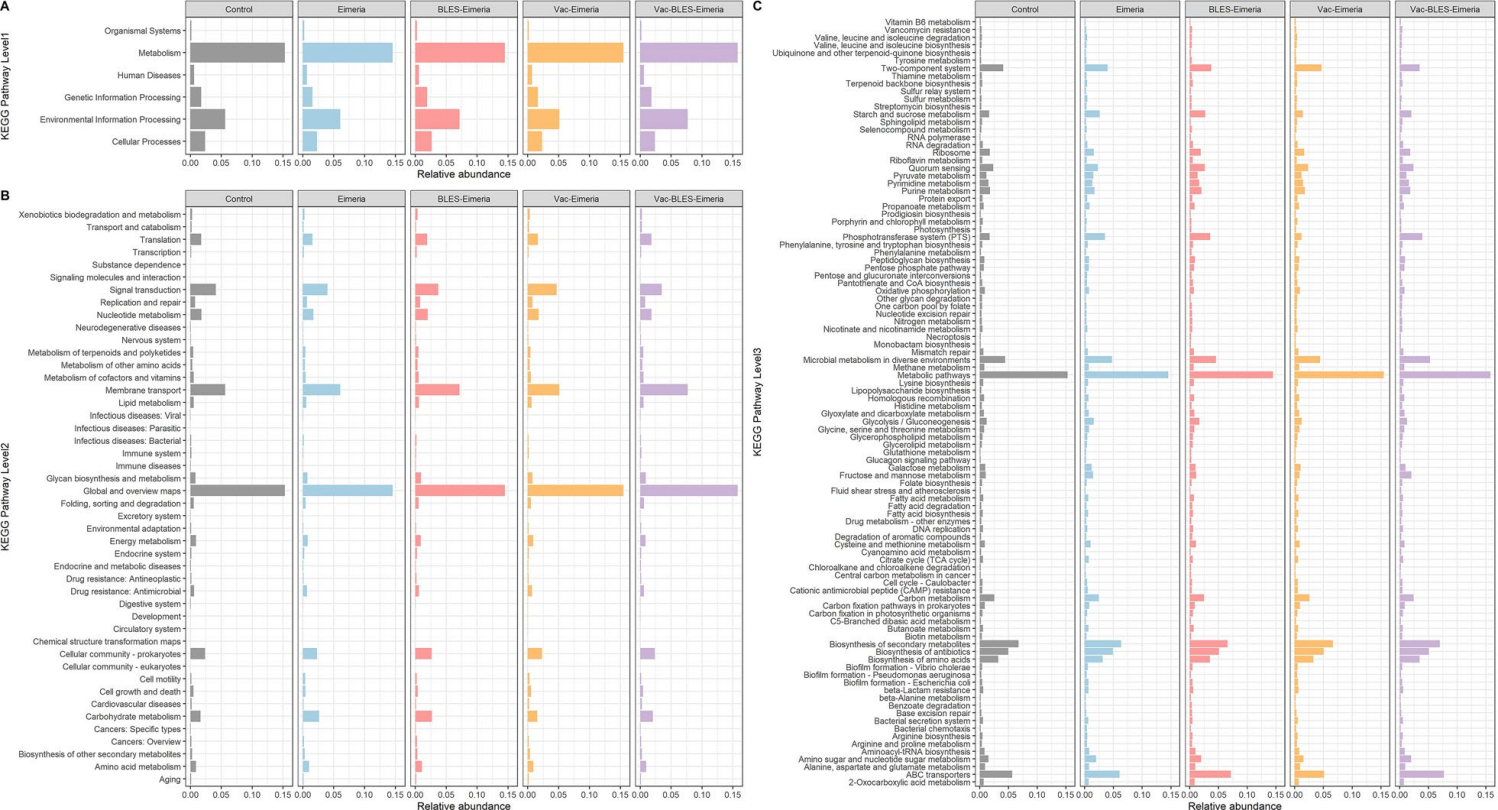
Prediction results of signaling pathways involved in the intestinal flora of each experimental group in the *Eimeria* spp. challenge experiment. The Tax4Fun assignment was utilized to predict the function of the intestinal flora and was used to classify KEGG pathway identification according to three levels: Level 1 (**A**), level 2 (**B**), and level 3 (**C**). The prediction was performed by cal_tax4fun2 function in R package microeco. KEGG, Kyoto Encyclopedia of Genes and Genomes

**Fig. 7:**
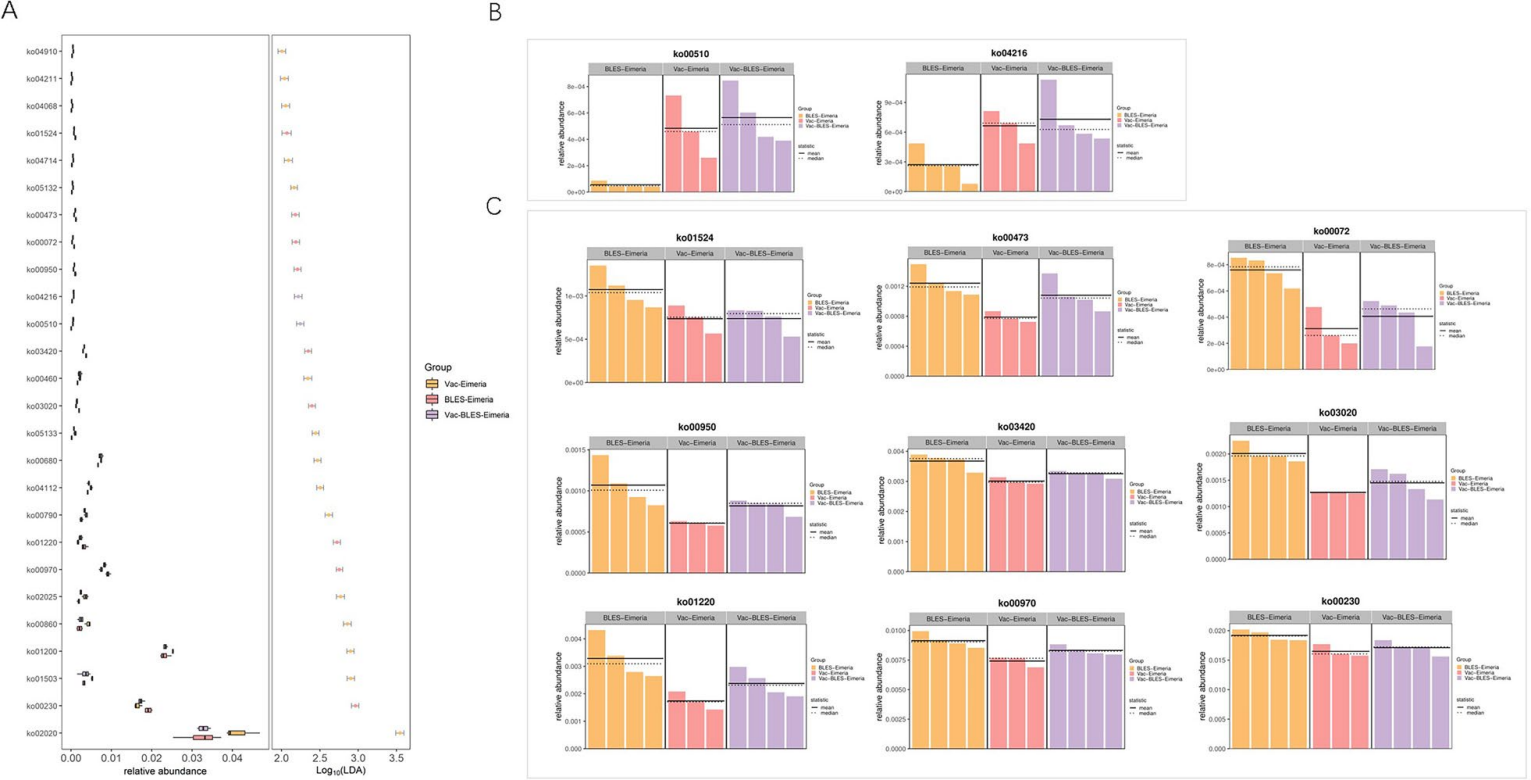
Results of the difference analysis for the intestinal flora function in the *Eimeria* spp. challenge experiment. **A** The LDA index was used to analyze the significance of the intestinal flora results.** B**,** C** Dominant signaling pathways of the Vac-BLES-Eimeria and the BLES-Eimeria are displayed in the form of histograms. Differences in molecular functions were analyzed by the LEfSe using diff_analysis function and plot built with ggdiffbox and ggdifftaxbar function in MicrobiotaProcess package (v.3.6.0). BLES, Fengqiang Shengtai quadruple probiotic preparation; Eimeria,* Eimeria *spp., LDA, linear discriminant analysis; Vac, vaccine against avian coccidiosis

Changes in the function of the microbial community in the challenge experiment were also analyzed. Five test groups in the challenge experiment were used for function prediction through the Tax4Fun software, and functional protein clustering was performed according to KEGG Level 1, Level 2 and Level 3. Overall, there were six functions in KEGG Level 1, among which the metabolism-related functions were the most abundant in the functional microbes (Fig. 6A). Within the KEGG Level 2 classification, 47 functional or enriched units were identified, among which the global and overview maps (a special class of metabolic pathway map) were the taxa with the most concentrated functional microbes (Fig. 6B). At KEGG Level 3, there were 327 signaling pathways that dynamically changed in the intestinal microbiota of the entire challenge experiment. Among these, the metabolic pathways, the ABC transporters, the biosynthesis of secondary metabolites and microbial metabolism in diverse environments had the highest enrichment levels (Fig. 6C). The LeFSe analysis method was applied to analyze the dominant signal pathway of the challenge experiment. The results of the LeFse analysis (LDA > 2 and *P* value < 0.05) showed that there were 26 signal pathways in the Vac-BLES-Eimeria group, the Vac-Eimeria group and the BLES-Eimeria group with significant differences (*P* < 0.05) (Fig. 7A). Among these, the Vac-BLES-Eimeria group mainly used N-glycan biosynthesis (KEGG pathway: ko00510) and ferroptosis (ko04216) as key signaling pathways (Fig. 7B), whereas, the BLES-Eimeria group used aminoacyl-tRNA biosynthesis (ko00970), nucleotide excision repair (ko03420), RNA polymerase (ko03020), synthesis and degradation of ketone bodies (ko00072), isoquinoline alkaloid biosynthesis (ko00950), D-alanine metabolism (ko00473), degradation of aromatic compounds (ko01220), purine metabolism (ko00230) and platinum drug resistance (ko01524). These nine signaling pathways were enriched in the BLES-Eimeria group (*P* < 0.05) (Fig. 7C).

## Discussion

The issue of drug resistance and residues of avian coccidiosis has received considerable critical attention in recent years. Research on alternative methods to control avian coccidiosis is thus of great significance in the poultry industry. At present, probiotics have been supplemented to animal feed as a means to treat various intestinal diseases and play a beneficial role in maintaining optimal animal health. Ritzi et al. [20] found that treatment with multi-probiotic strains during an *Eimeria* infection had the capacity to increase BWG and to reduce oocyst excretion and the severity of intestinal lesions. The addition of probiotics to an anti-coccidian drug formulaiton has been proven to be effective in improving oocyst shedding and the lesion score. In addition, the use of probiotics or the combined use of probiotics with antibiotics for the treatment of *Eimeria* spp. infection has been shown to have some preventive effect [21–23]. In the present study we continued our exploration of the functions of the probiotic product Fengqiang Shengtai feed additive (BLES) and live avian coccidiosis vaccine in broiler production performance, intestinal health and prevention of chicken coccidiosis. The aim was to improve our understanding of the microbiome of the immune strategy by comparing the structure and function of the intestinal microbial community in the immune stage and the *Eimeria* spp. challenge stage.

The results of the current study revealed that the combined application of the probiotic product Fengqiang Shengtai and avian coccidiosis live vaccine yields significant improvements in the production performance of broiler chickens. Coccidiosis leads to substantial economic losses in broiler production, primarily due to increased drug costs and lower production performance [24]. While live avian coccidiosis vaccine can effectively prevent and control avian coccidiosis, it may also negatively impact early growth performance in broilers and potentially increase the susceptibility of broilers to secondary infection [9]. Therefore, effectively improving the production performance of broiler chickens becomes crucial to fully leverage the advantages of the avian coccidiosis live vaccine in the prevention of coccidia infection. Our findings demonstrated that treatment with the combination of BLES and live avian coccidiosis vaccine during the finisher period significantly reduces FI and FCR values (Fig. 1C, D), thereby improving the feed conversion efficiency. BLES is a quadruple live bacterial preparation composed of *Bifidobacterium*, *Lactobacillus*, *Streptococcus faecalis* and yeast. Ritzi et al. previously reported that when the Poultrystar multi-species symbiotic product (*Bifidobacterium animalis*, *Lactobacillus salivarius*, *Enterococcus faecium*) and Immucox I vaccine were administered together, the FI and FCR of broilers were significantly lower than those immunized with avian coccidiosis live vaccine alone [25]. In Ritzi et al.’s study [25], the probiotics were administered on alternate days until 36 days of age of the chicken, with the challenge performed at 15 days of age. However, in our study, the finisher period was considered to be optimal timing for BLES administration, which was a specific choice. By this stage, the chickens had entered a stable growth phase, and the administration of BLES was expected to yield a more significant effect on production performance. This approach was selected to enhance the reliability and robustness of our findings. Overall, our results are consistent with those of previous studies, suggesting that the combined use of probiotics and live avian coccidiosis vaccine can effectively ameliorate the decrease in production performance associated with the use of live avian coccidiosis vaccine.

Oocyst shedding and the lesion score are commonly used clinical indicators to assess the severity of coccidiosis. The combined application of BLES and live avian coccidiosis vaccine resulted in a significant reduction in disease indicators associated with avian coccidiosis. In this study, the group infected solely with *Eimeira* spp. displayed a significant increase in OPG value and lesion score (*P* < 0.05) (Fig. 1E, F), while the combined administration of BLES and live avian coccidiosis vaccines resulted in a significant decrease in the OPG and lesion scores (*P* < 0.05). The results clearly demonstrate the effectiveness of the combined application strategy of BLES and live avian coccidiosis vaccine in preventing and controlling chicken coccidia infection. Notably, previous studies also reported similar findings, such as the co-administration of the PoultryStar and the Immucox I vaccine, which led to a reduction in intestinal lesion scores [25]. In addition, the changes in intestinal pathology in tissues from chickens subjected to different immune strategies during avian coccidiosis infection were evaluated. In one study, *Eimeria* infection was shown to be capable of downregulating mRNA expression of occludin and claudin-1 [26]. These tight junction proteins are closely related to intestinal permeability in chickens [27]. Previous studies have also reported that *Eimeria* spp. induces damage to chicken epithelial cells, leading to the detachment of intestinal epithelial cells from epithelial villi. The damage appears to be closely linked to its disruption of tight junction proteins [28]. Our results showed that the combined application of BLES and the live avian coccidiosis vaccine did not cause obvious intestinal epithelial damage (Fig. 2E). Numerous studies have reported the potential of probiotics and their bioactive compounds to modulate the intestinal barrier and mucosal immunity through various mechanisms, including regulating mucus production, reducing bacterial adhesion, enhancing tight junctions and cell survival and inducing cytokines [29, 30]. In this context, it is reasonable to speculate that BLES may also regulate the expression of tight junction proteins through the secretion of bioactive compounds, which in turn play a protective role in the intestinal barrier. However, further experiments are needed to validate the underlying mechanisms.

Intestinal health is intricately linked to the interaction between the host and the microbiota. The microbiota plays a pivotal role in regulating nutrient absorption efficiency, countering the influence of pathogenic bacteria, enhancing intestinal integrity and modulating immune response. In the context of coccidiosis infection, various alterations occur, including changes in nutrient absorption and digestibility, increased mucosal formation, increased membrane permeability, increased nutrient availability and the proliferation of pathogenic bacteria. Vieira et al. observed that *Eimeria* spp. infection resulted in an increased abundance of *Erysipelotrichaceae*, *Lactobacillus*, *Bacteroidaceae*, *Streptococcaceae* and *Peptostreptococcaceae*, while reducing the population of *Ruminococcaceae* [31]. Chen et al. [32] previously reported dynamic shifts in the cecal microbial community following *Eimeria* spp. infection, including reduction in *Lactobacillus*, *Faecalibacterium*, *Ruminococcaceae* UCG-013, *Romboutsia,* and *Shuttleworthia*; furthermore, opportunistic pathogens, such as *Enterococcus* and *Streptococcus*, increased in abundance over time in response to the infection. Nevertheless, there is still limited information on the role of chicken intestinal microbiota and its response to the vaccination under different feeding schemes.

We found that the *Lachnospiraceae* NK4A136 group was the representative bacterial genus of the combined application strategy of BLES and the avian coccidiosis vaccine at the 15-day-old stage. The *Lachnospiraceae* NK4A136 group is a butyrate-producing probiotic that has previously been reported to improve gut barrier function during aging [33]. In addition, in the *Eimeria* spp. challenge experiment of our study, the high-abundance ASVs that were unique to the VAC-BLES-Eimeria group were concentrated in six genera: *Olsenella*, *Barnesiella*, *Bacteroides*, *Subdoligranulum*, *Anaerotruncus* and *Blautia*, and the low-abundance ASVs were concentrated in *Escherichia, Shigella* and *Enterococcus*. *Escherichia*,* Shigella* and *Enterococcus* are opportunistic pathogens [34]. The negative regulation of these bacterial genera in the VAC-BLES-Eimeria group might help to ensure that the stability of the intestinal microbiota is maintained. Although our study offers important insights into the changes in the intestinal microbiota following the combined application strategy, the relationship between these changes and broiler performance remains unclear and warrants further investigation. Subsequent studies could focus on elucidating the potential connections between the abundance of specific bacterial genera and the indicators of broiler growth performance, including FCRs and BWG.

To further utilize taxonomic abundance data, this study performed Tax4Fun analysis to determine the functional characteristics of the microbiota that may contribute to disease resistance. The findings of this analysis provided evidence that the combined administration of BLES and the live avian coccidiosis vaccine exerts a significant impact on the composition of the microbiota involved in N-glycan biosynthesis (KEGG pathway: ko00510) and ferroptosis (ko04216) (Fig. 7B). Glycosylation is a prevalent post-translational modification in organisms that is observed in over half of the proteins examined to date [35]. In eukaryotic cells, N-glycosylation has emerged as the primary form of glycosylation, with the diversity and branching patterns of N-glycans playing a crucial role in regulating essential cellular processes, such as cell growth, differentiation and, in particular, the immune recognition system [36]. Previous studies have documented that lectins derived from *Paracoccidioides brasiliensis* and *Toxoplasma gondii* interact with Toll-like receptor (TLR) 2 and the TLR4 N-glycans, thereby conferring protection against the corresponding infections [37, 38].

The immune recognition response observed in this study was closely associated with the N-glycan biosynthesis pathway, suggesting that the combined strategy employed in this study can effectively stimulate the production of N-glycans, potentially leading to enhanced protection against infections through the activation of immune recognition responses. While previous studies have highlighted the involvement of N-glycosylation in regulating crucial processes related to cell growth and differentiation, our findings provide new insights into the potential contribution of this pathway in augmenting disease resistance in broilers. Moreover, it is important to note ferroptosis, which is an iron-dependent form of cell death that differs from apoptosis, necrosis, autophagy and other forms of cell death [39]. Kain et al. [40] reported that ferroptosis could be regulated by the SLC7a11-GPX4 signaling pathway to inhibit *Plasmodium* liver-stage infection. Hence, in our study, we employed the Tax4Fun analysis method to determine the functional characteristics of the microbiota involved in N-glycan biosynthesis and ferroptosis processes, with the results suggesting their potentially critical role in enhancing disease resistance. However, to further expand and refine our findings, additional experiments are necessary.

## Conclusions

In conclusion, the combined administration of BLES alongside a live avian coccidiosis vaccine yielded an substantial enhancement of broiler production performance and a notable alleviation of clinical lesions associated with chicken coccidiosis. Our findings suggest that the efficacy of this strategy is closely tied to its modulation of the intestinal microbiota, particularly by suppressing the abundance of the opportunistic pathogens such as *Escherichia*,* Shigella* and *Enterococcus.* Furthermore, we identified the involvement of key signaling pathways, namely N-glycan biosynthesis (KEGG pathway: ko00510) and Ferroptosis (ko04216), in facilitating the observed protective effects.

## Supplementary Information

**Additional file 1: Table S1.** Tests of between-subjects effects in Fengqiang Shengtai feed additive (BLES) and tetravalent live vaccine (Vac) for Performance indicators. **Figure S1**. Rarefaction curves and alpha diversity plots of each sample group. (A) Rarefaction curve constructed based on observed ASVs. The curve analyzed by get_rarecurve function in R package MicrobiotaProcess (v.3.6.0). (B)–(D) Box plot of Chao index, Shannon index and Simpson index for each group in day-8, day-15 and day-32. The alpha diversity index were calculated by get_alphaindex function. **Figure S2.** Beta diversity shown by principal coordinates analysis (PCoA) of bray-curtis (left) and Euclidean distances (right) by using get_pcoa function in MicrobiotaProcess package. (A)–(C) represented the PCoA results for the treatment groups at different ages. Biplots display the taxonomy of the ASVs with the top 5 effects on the community composition as can be identified with the position of the arrow, which indicated the direction of the effect. The Principal Coordinate Analysis (PCoA) plot built by using ggordpoint in MicrobiotaProcess package.

## Data Availability

The raw sequencing data for bacterial communities have been submitted to the National Center of Biotechnology Information (NCBI) Sequence Read Archive under accession number PRJNA823896.

## Abbreviations

ASVs: Amplicon sequence variants
BLES: Fengqiang shengtai (*Bifidobacterium animalis*, *Lactobacillus casei*, *Enterococcus faecalis* and *Saccharomyces cerevisiae*)
BWG: Body weight gain
CFU: Colony-forming unit
FCR: Feed conversion ratio
FI: Feed intake
H&E: Hematoxylin and eosin stain
KEGG: Kyoto Encyclopedia of Genes and Genomes
LDA: Linear discriminant analysis
LEfSe: LDA effect size
OPG: Oocysts per gram
PCoA: Principal coordinate analysis
rRNA: Ribosomal RNA
TLR: Toll-like receptor
QIIME2: Quantitative insights into microbial ecology 2
