# Supplementary material for: Effects of a complex probiotic preparation, Fengqiang Shengtai and coccidiosis vaccine on the performance and intestinal microbiota of broilers challenged with Eimeria spp
Source: Parasit Vectors. 2023 Jul 27;16:253. doi: 10.1186/s13071-023-05855-5 (PMC10375739; doi:10.1186/s13071-023-05855-5)
Supplement: Supplementary file 1 — Additional file 1: Table S1. Tests of between-subjects effects in Fengqiang Shengtai feed additive (BLES) and tetravalent live vaccine (Vac) for Performance indicators. Figure S1. Rarefaction curves and alpha diversity plots of each sample group. (A) Rarefaction curve constructed based on observed ASVs. The curve analyzed by get_rarecurve function in R package MicrobiotaProcess (v.3.6.0). (B)–(D) Box plot of Chao index, Shannon index and Simpson index for each group in day-8, day-15 and day-32. The alpha diversity index were calculated by get_alphaindex function. Figure S2. Beta diversity shown by principal coordinates analysis (PCoA) of bray-curtis (left) and Euclidean distances (right) by using get_pcoa function in MicrobiotaProcess package. (A)–(C) represented the PCoA results for the treatment groups at different ages. Biplots display the taxonomy of the ASVs with the top 5 effects on the community composition as can be identified with the position of the arrow, which indicated the direction of the effect. The Principal Coordinate Analysis (PCoA) plot built by using ggordpoint in MicrobiotaProcess package. [file 13071_2023_5855_MOESM1_ESM.docx]

**Supplementary Material**

**Effects of a complex probiotic preparation, Fengqiang Shengtai and coccidiosis vaccine on performance and intestinal microbiota of broilers challenged with *Eimeria* spp.**

Haiming Cai^1#^, Shengjun Luo^1#^, Qihong Liu^2^, Qingfeng zhou^3^, Zhuanqiang Yan^3^, Zhen Kang^4^, Shenquan Liao^1^, Juan Li^1^, Minna Lv^1^, Xuhui Lin^1^, Junjing Hu^1^, Shuilan Yu^3^, Jianfei Zhang^1^, Nanshan Qi^1*^, Mingfei Sun^1*^

1 Zhaoqing/Maoming Branch Center of Guangdong Laboratory for Lingnan Modern Agricultural Science and Technology; Key Laboratory of Livestock Disease Prevention of Guangdong Province; Key Laboratory of Avian Influenza and Other Major Poultry Diseases Prevention and Control, Ministry of Agriculture and Rural Affairs; Institute of Animal Health, Guangdong Academy of Agricultural Sciences, Guangzhou 510640, Guangdong, P. R. China.

2 Jiangsu HFQ Biotechnology Co., Ltd., Haimen, Jiangsu Province, People’s Republic of China.

3 Wen's Group Academy, Wen's Foodstuffs Group Co., Ltd., Xinxing, Guangdong 527400, China.

4 Qingdao Vland Biotech Group Co., Ltd., Shandong Province, People’s Republic of China.

# These authors contributed equally.

*Correspondence: Sun Mingfei, [smf7810@126.com](mailto:smf7810@126.com); Qi Nanshan, [nanshanqi@163.com](mailto:nanshanqi@163.com).


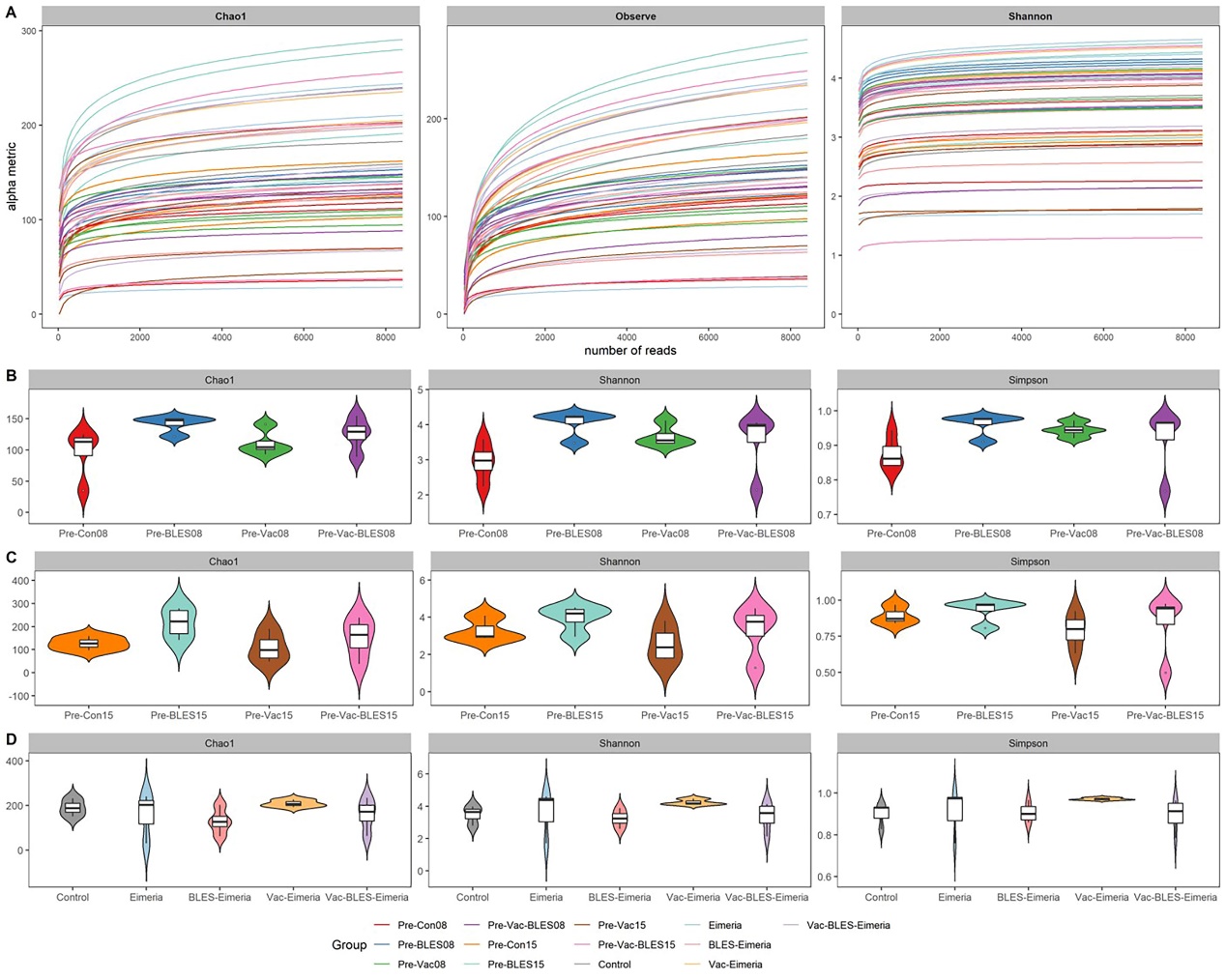


**Fig. S1**. Rarefaction curves and alpha diversity plots of each sample group. (A) Rarefaction curve constructed based on observed ASVs. The curve analyzed by get_rarecurve function in R package MicrobiotaProcess (v.3.6.0). (B)-(D) Box plot of Chao index, Shannon index and Simpson index for each group in day-8, day-15 and day-32. The alpha diversity index were calculated by get_alphaindex function.


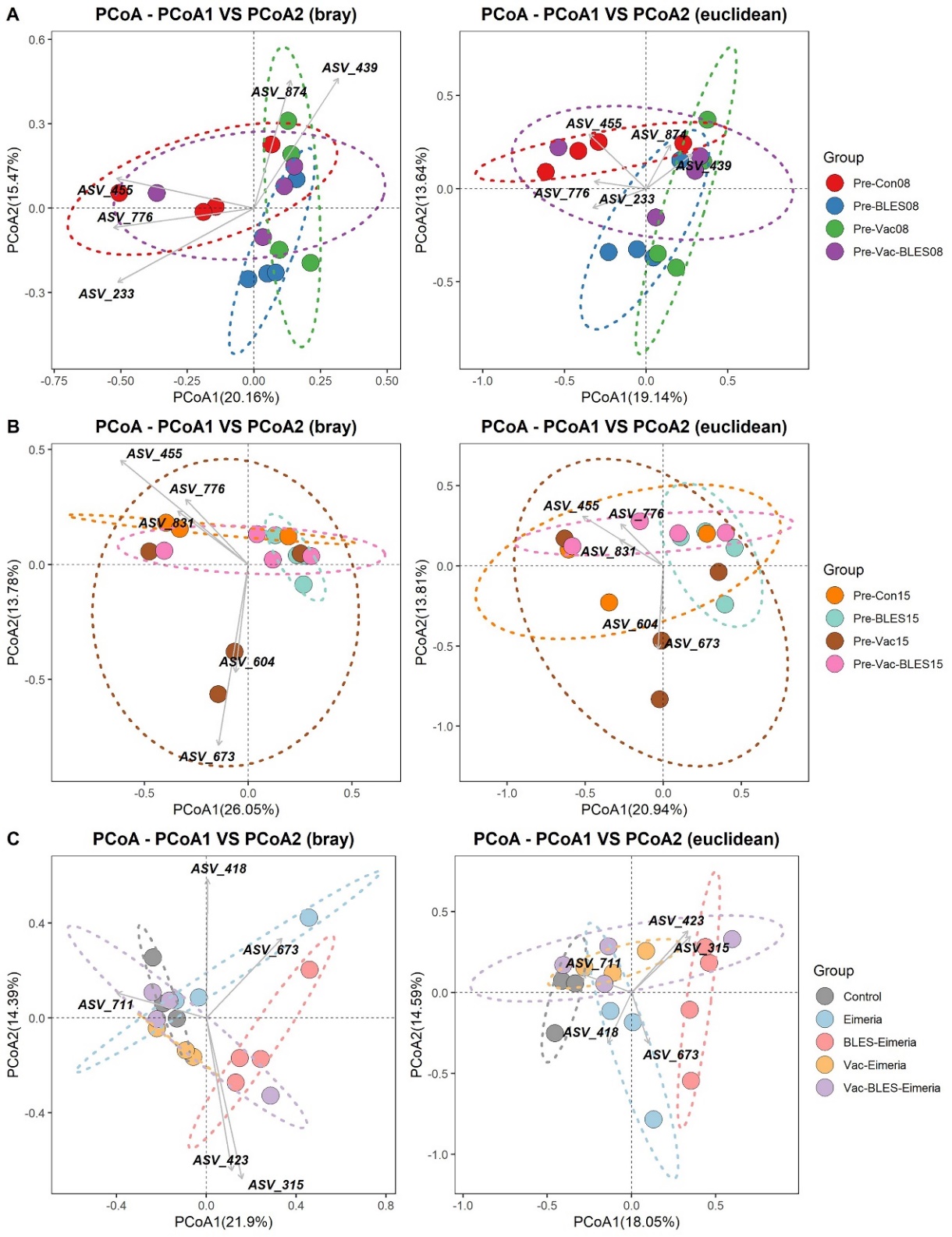


**Fig. S2**. Beta diversity shown by principal coordinates analysis (PCoA) of bray-curtis (left) and Euclidean distances (right) by using get_pcoa function in MicrobiotaProcess package. (A)-(C) represented the PCoA results for the treatment groups at different ages. Biplots display the taxonomy of the ASVs with the top 5 effects on the community composition as can be identified with the position of the arrow, which indicated the direction of the effect. The Principal Coordinate Analysis (PCoA) plot built by using ggordpoint in MicrobiotaProcess package.

**Table S1**. Tests of between-subjects effects in Fengqiang Shengtai feed additive (BLES) and tetravalent live vaccine (Vac) for Performance indicators

|  | Body weight gain | | | Feed intake | | | Feed conversion ratio | | |
| --- | --- | --- | --- | --- | --- | --- | --- | --- | --- |
|  | F | Sig. | partial η2 | F | Sig. | partial η2 | F | Sig. | partial η2 |
| BLES | 1.840 | 0.228 | 0.345 | 7.595 | 0.024 | 0.859 | 17.076 | 0.004 | 0.932 |
| Vac | 233.720 | 0.000 | 0.985 | 22.664 | 0.002 | 0.948 | 78.240 | 0.000 | 0.984 |
| BLES + Vac | 266.260 | 0.000 | 0.987 | 13.167 | 0.007 | 0.913 | 30.849 | 0.001 | 0.961 |
